# Functions and regulation of quorum-sensing in *Agrobacterium tumefaciens*

**DOI:** 10.3389/fpls.2014.00014

**Published:** 2014-01-31

**Authors:** Julien Lang, Denis Faure

**Affiliations:** Institut des Sciences du Végétal, UPR2355, Centre National de la Recherche ScientifiqueGif-sur-Yvette, France

**Keywords:** quorum-sensing, opines, conjugation, genetic, plant host, quorum-quenching, gene expression regulation

## Abstract

In *Agrobacterium tumefaciens*, horizontal transfer and vegetative replication of oncogenic Ti plasmids involve a cell-to-cell communication process called quorum-sensing (QS). The determinants of the QS-system belong to the LuxR/LuxI class. The LuxI-like protein TraI synthesizes *N*-acyl-homoserine lactone molecules which act as diffusible QS-signals. Beyond a threshold concentration, these molecules bind and activate the LuxR-like transcriptional regulator TraR, thereby initiating the QS-regulatory pathway. For the last 20 years, *A. tumefaciens* has stood as a prominent model in the understanding of the LuxR/LuxI type of QS systems. A number of studies also unveiled features which are unique to *A. tumefaciens* QS, some of them being directly related to the phytopathogenic lifestyle of the bacteria. In this review, we will present the current knowledge of QS in *A. tumefaciens* at both the genetic and molecular levels. We will also describe how interactions with plant host modulate the QS pathway of *A. tumefaciens*, and discuss what could be the advantages for the agrobacteria to use such a tightly regulated QS-system to disseminate the Ti plasmids.

## INTRODUCTION

In its canonical definition, quorum-sensing (QS) refers to a process through which a bacterial population is able to monitor its cell density and accordingly to mount coordinate responses (Fuqua et al., 1994). This phenomenon relies on the synthesis, diffusion, and perception of small signal molecules (autoinducers) that allow bacteria to communicate with each other and to regulate gene expression. In the last 40 years, a number of studies have established that QS is widespread in the bacterial kingdom although the nature of the signal molecules and/or signaling networks as well as the functions regulated by QS may vary considerably depending on the species (Miller and Bassler, 2001; Frederix and Downie, 2011; Stevens et al., 2012; Pereira et al., 2013).

In Proteobacteria, the typical QS model is epitomized by the LuxI/LuxR bioluminescence system of *Vibrio fischeri* that was described as early as 1970 (Nealson et al., 1970; Eberhard, 1972). In summary, LuxI catalyzes the synthesis of an *N*-acyl-homoserine lactone, namely the 3-oxo-hexanoyl-homoserine lactone (3OC6HSL), that acts as an autoinducer and accumulates in a cell density-dependent manner. At a threshold concentration, the 3OC6HSL molecules bind to their ligands, the transcriptional factor LuxR, and the newly formed LuxR dimers induce the expression of the *lux* operon which includes the genes responsible for bioluminescence but also *luxI*. This last autoregulatory action results in an exponential increase of the production of autoinducers and accounts for the characteristic pattern of QS-dependent bioluminescence in *V. fischeri* populations which rapidly shift at the quorum concentration from an “off” state to an “on” state.

Interestingly many homologs of LuxI and LuxR proteins have been found in other bacterial species such as *Pseudomonas aeruginosa*, *Pectobacterium atrosepticum*, and *Agrobacterium tumefaciens* (Fuqua et al., 1994, 1996). The first milestone in the study of *A. tumefaciens* QS was the functional characterization of the TraR protein, the LuxR homolog (Piper et al., 1993; Zhang et al., 1993). This seminal finding opened a new area of research in horizontal transfer of virulence Ti plasmids in *A. tumefaciens* that made this phytopathogenic species a leading model for the investigation of LuxI/LuxR QS systems. In this review, we will recap the most striking results obtained in deciphering the genetic network as well as the molecular basis of *A. tumefaciens* QS. We will also present how this QS system, consistent with the phytopathogenic lifestyle of *A. tumefaciens*, is integrated into an exquisite regulatory process, including various opine-induced regulons and lactonase activities. Finally we will discuss the biological/evolutionary relevance of this complex network in terms of dissemination of Ti plasmid genes in the plant tumor environment.

## OVERVIEW OF *A. tumefaciens* QS

### A LuxI/LuxR TYPE QS INTEGRATING AN ANTAGONIST COMPONENT

**FIGURE 1:**
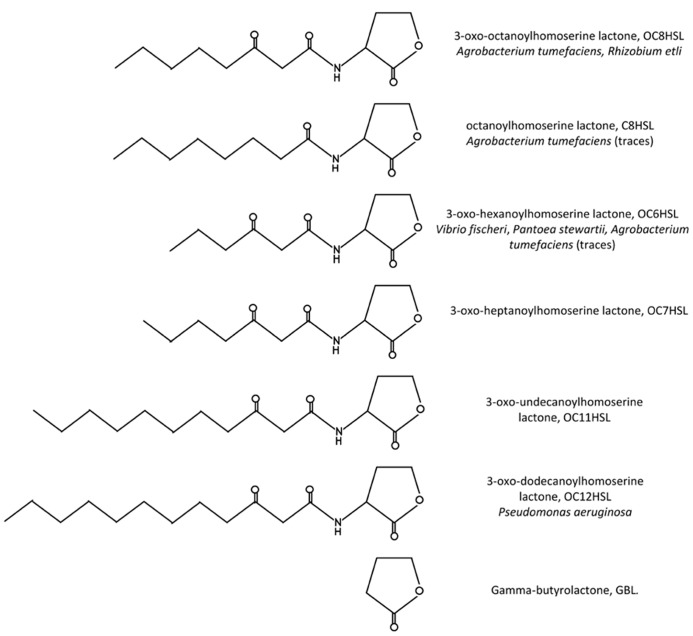
**Structures of the QS signals mentioned in this review.** In parallel are indicated the full name of the molecule, its abbreviation as well as some bacterial species known to produce it.

The first insight of a QS system in *A. tumefaciens* was gained with the functional characterization of a *traR* gene, homologous to *V. fischeri luxR*, the product of which acted as a transcriptional activator in the presence of a co-inducer. Actually two versions of the *traR* gene were found almost concomitantly in nopaline- and octopine-type Ti plasmids (Piper et al., 1993; Fuqua and Winans, 1994). These genes displayed high homology between them but were located in dissimilar regions of the two Ti plasmids, the expression of each of these regions being controlled by specific opines. Along with these discoveries, the chemical structure of the co-inducer required for TraR activity was determined by spectrometry analysis as 3-oxo-octanoyl-homoserine lactone (OC8HSL, see structure in **Figure 1**; Zhang et al., 1993). Soon afterward the gene *traI*, for which very closely related sequences also exist in nopaline- and octopine-type Ti plasmids, was shown to be responsible for OC8HSL synthesis (Hwang et al., 1994).

Like other LuxI/LuxR type QS systems, *A. tumefaciens* QS comprises another component that negatively modulates the activity of TraR and OC8HSL and this component is the Ti plasmid-encoded protein TraM which can suppress TraR transcriptional activity. Versions of the *traM* gene were identified in both nopaline- and octopine-type Ti-plasmids (Fuqua et al., 1995; Hwang et al., 1995). The octopine-type Ti plasmid A6 even possesses a second functional *traM* gene borne on a chromosome, surely as a result of gene duplication (Wang et al., 2006a). For long it has been thought that TraM proteins were not related to any other proteins found in the databases, but recent characterization of the *Pseudomonas aeruginosa* QslA protein contradicted this view (Seet and Zhang, 2011), suggesting that TraM-type functions might be relatively common in bacteria.

At a mechanistic level, yeast two-hybrid assays revealed that TraM and TraR could directly interact. From these data it was deduced that the association between the two proteins was responsible for the inhibition of TraR-mediated responses by preventing proper TraR binding to DNA (Hwang et al., 1999). Two subsequent findings strengthened the negative regulatory functions exerted by TraM on QS. First it was established that this protein could block TraR activity even after the transcription factor has bound to DNA (Luo et al., 2000) and second TraM was demonstrated to promote TraR proteolysis (Costa et al., 2012).

The implications of TraM action for the dynamics of the QS system will be discussed in the following section.

### QS-REGULATED GENES ARE INVOLVED IN FEEDBACK CONTROL AND Ti PLASMID DISSEMINATION

**FIGURE 2:**
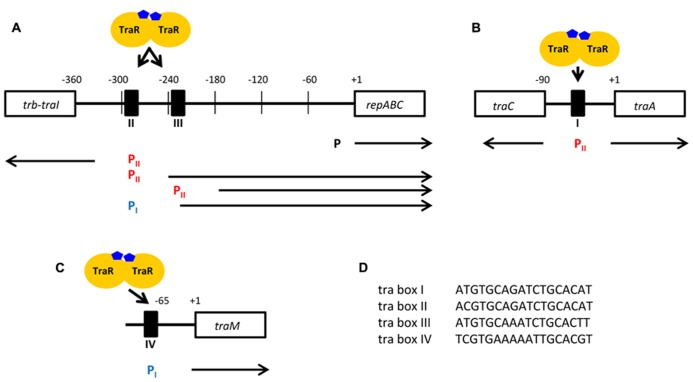
**Promoter architecture of the TraR-regulated genes in *A. tumefaciens*. (A–C)** Representation of the regions upstream of the *traI-trbBCDEJKLFGHI* and *repABC* operons **(A)**, *traCDGyci* and *traAFBH* operons **(B)** *traM* gene **(C)**. The tra boxes (I, II, III, and IV) are indicated by black boxes. Under each tra box are presented the associated promoters, the activations of which are dependent on the binding of TraR. The promoters of class I-type are in blue while those of class II-type are in red. The fourth identified promoter controlling the expression of *repABC* in a TraR-independent way is also displayed (P). The arrows indicate the direction of transcription. **(D)** The nucleotide sequences of the four tra boxes. (Adapted from Fuqua and Winans, 1996a; Pappas and Winans, 2003a,b; White and Winans, 2005).

Chronologically the first TraR-regulated, hence QS-regulated, genes were the OC8HSL synthesis *traI* gene and the *tra* genes involved in conjugation of the Ti plasmid (Piper et al., 1993; Fuqua and Winans, 1994; Hwang et al., 1994). Next, were the regulatory gene *traM* (Fuqua et al., 1995; Hwang et al., 1995) and finally the *rep* genes required for vegetative replication of the Ti plasmid (Li and Farrand, 2000). Concomitantly, four 18 bp-inverted repeat operator sequences (called tra box I, II, III, and IV), the disruption of which abolished the TraR transactivation, were found in the promoter regions of the QS-regulated genes. These promoters were assigned to two distinct classes (class I-type and class II-type) according to the position of the tra boxes relatively to the transcription initiation site. In promoters of class I-type, the tra box is located approximately 65 nucleotides upstream of the transcription start site and in promoters of class II-type, the tra box is located about 45 nucleotides upstream of the transcription start site, partially overlapping with the -35 element of the promoter (**Figure 2**; Fuqua and Winans, 1996a). The *traR* gene has also been reported as being self-regulated though no tra box was detected in its promoter region (Fuqua and Winans, 1996b).

In line with the above studies, an extensive survey of QS-regulated genes has been recently carried out both in nopaline- and octopine-type Ti plasmids, using gene arrays and a TraR-overexpressing system (Cho and Winans, 2007). The results globally confirmed the previous data. Only genes located in the Ti plasmids were affected. In nopaline-type Ti plasmid, 31 genes were up-regulated in response to TraR overexpression and 25 in octopine-type Ti plasmid. Among the up-regulated genes common to the two plasmids, were the *tra*, *rep*, and *traM* genes. Moreover the operon structures, the presence of tra boxes in the promoter regions and the overall regulation of the expression of these genes were well conserved within the two plasmids.

**Table 1** summarizes the identities and functions of the *A. tumefaciens* QS-regulated genes which are detailed in the following. The *traCDGyci* and *traAFBH* operons are divergently transcribed from a single class II-type promoter activated by a tra box I. These genes code for a DNA transfer and replication machinery involved in the conjugative processing of the Ti plasmid (Farrand et al., 1996; Cook et al., 1997; Cho and Winans, 2007). The proteins TraA, TraC, and TraD are notably thought to form a relaxosome at the oriT of the Ti plasmid which can also repress the expressions of both *traCDGyci* and *traAFBH* operons (Cho and Winans, 2007). The promoter of *traI-trbBCDEJKLFGHI* operon belongs to the class II-type of QS-regulated promoter but is characterized by the presence of a tra box II. The *trb* genes encode a mating pair formation system for the transfer of the Ti plasmid which is related to type IV secretion systems (Li et al., 1998). Among the proteins encoded by these genes, TrbJ and TrbK also act synergistically to implement an entry exclusion mechanism which ensures that conjugation events cannot occur between donor and recipient *A. tumefaciens* cells harboring similar Ti plasmids (Cho et al., 2009). In agreement with the gene functions, TraR-mediated up-regulation of the three *traCDGyci*, *traAFBH* and *traI-trbCDEJKLFGHI* operons results in induction of Ti plasmid conjugation. On the other hand the control of *traI* expression by TraR leads to a positive feedback loop which amplifies, through increase in OC8HSL production, the QS responses of *A. tumefaciens* (Fuqua and Winans, 1994; Hwang et al., 1994). As an illustration of this effect, exogenous supply of OC8HSL to *A. tumefaciens* cells accelerated the TraR-mediated induction of Ti plasmid conjugation (Fuqua and Winans, 1996a).

**Table 1:** List of QS-regulated genes in nopaline- and octopine-type Ti plasmids (adapted from Cho and Winans, 2007).

Gene name	Function	atu code
*traC*	Conjugal transfer protein	*atu6126*
*traD*	Conjugal transfer protein	*atu6125*
*traG*	Conjugal transfer protein	*atu6124*
*yci*	Nuclease	*atu6122*
*traA*	Conjugal transfer protein	*atu6127*
*traF*	Conjugal transfer protein	*atu6128*
*traB*	Conjugal transfer protein	*atu6129*
*traH*	Conjugal transfer protein	*atu6130*
*traI*	Acyl-homoserine-lactone synthase	*atu6042*
*trbB*	Conjugal transfer protein	*atu6041*
*trbC*	Conjugal transfer protein	*atu6040*
*trbD*	Conjugal transfer protein	*atu6039*
*trbE*	Conjugal transfer protein	*atu6038*
*trbJ*	Conjugal transfer protein	*atu6037*
*trbK*	Entry-exclusion protein	*atu6036*
*trbL*	Conjugal transfer protein	*atu6035*
*trbF*	Conjugal transfer protein	*atu6034*
*trbG*	Conjugal transfer protein	*atu6033*
*trbH*	Conjugal transfer protein	*atu6032*
*trbI*	Conjugal transfer protein	*atu6031*
*traM*	Transcriptional anti-activator	*atu6131*
*repA*	Plasmid-partitioning protein	*atu6043*
*repB*	Plasmid-partitioning protein	*atu6044*
*repC*	Replication initiation protein	*atu6045*

Curiously the *traM* gene coding for the TraR antiactivator appears also to be up-regulated by TraR (Hwang et al., 1995). It was proposed that this regulatory mechanism allows the cells to produce TraM proteins at levels sufficient to inhibit the available TraR under conditions of basal-level expression. Later on, when the expression of *traR* is induced, the resulting increased levels of TraR protein would overcome the available TraM, thence triggering the QS response. This model actually highlights the importance of relative TraR and TraM protein levels in QS regulation and suggests that TraM significantly contributes to the quorum-dependent dimension of the system by delaying the moment when TraR is able to transactivate target genes (Su et al., 2008). Consistently, a *traM* defective strain was shown to be QS active in a cell density-independent manner (Piper and Farrand, 2000). Furthermore, a mathematical approach claimed that TraM was necessary for the existence of the *A. tumefaciens* QS “off” state (Goryachev et al., 2005). Another implication of the *traM* regulation by TraR is that the rate of TraR production must at one point exceed that of TraM production, otherwise QS activation would continuously be inhibited. Evidence that TraM is specifically transcribed from a mildly activated promoter with a tra box IV (White and Winans, 2005) is in line with this requirement. Alternatively an interesting but yet unexplored possibility to explain the induction of *traM* expression by TraR would be that this mechanism provides the cells with a mean to limit or shut off the QS process when this one is too strongly activated and becomes for instance too demanding energetically. This down-regulation loop is indeed common in other LuxI/LuxR systems (Gelencser et al., 2012). Either way a more critical examination of TraM regulation is still needed to fully clarify its role in QS. Additionally it has been shown that acetosyringone, a phenolic compound released by wounded plant cells, could also induce expression of *traM*, suggesting that during first steps of tumorigenesis TraM could efficiently inhibit QS activity (Cho and Winans, 2005).

The *A. tumefaciens* Ti-plasmids use an original system of replication and partitioning encoded in a single locus named *repABC*. While RepC is essential for replicative DNA synthesis, RepA and RepB are thought to be involved in stable partitioning of plasmids into daughter cells (Pinto et al., 2012). Initially the expression of the operon *repABC* was shown to be strongly stimulated by TraR in bacterial backgrounds with both nopaline- and octopine-type plasmids. This stimulation was also correlated with induction of vegetative replication, i.e., with a drastic increase in number of Ti plasmid copies per cell (Li and Farrand, 2000; Pappas and Winans, 2003a). However, in the array experiment mentioned previously (Cho and Winans, 2007), *repABC* up-regulation by TraR was barely detectable. The authors argued that this result was probably due to the very weak basal expression of the operon and that it did not question the role of QS in controlling the number of Ti plasmid copies because under their experimental conditions the number of Ti plasmids per cell was still higher than one. Another interpretation of this result might be that increased Ti plasmid copies culminate in a negative feedback control possibly bringing back the expression of the *repABC* genes to their basal levels, thereby avoiding continuous and anarchic replication of the replicon. The promoter architecture of *repABC* may support this hypothesis as three different TraR-dependent (repAP1, 2, and 3) and one TraR-independent (repAP4) promoters control the expression of the operon (Pappas and Winans, 2003b). Promoter repAP4 is thought to mediate the Ti plasmid replication associated with cell division but it is also autorepressed by RepA and RepB. Moreover repAP4 is located downstream of repAP1, 2, and 3. It is therefore conceivable that autorepression of repAP4 might impair activation of TraR-dependent promoters. Additionally expression of *repABC* can be induced by the virulence proteins VirA and VirG, further suggesting that the regulation of this operon is complex and might be sensitive to different physiological states (Cho and Winans, 2005; Pappas, 2008).

## MECHANISTIC INSIGHTS INTO *A. tumefaciens* QS

A central aspect of the LuxI/LuxR type QS systems resides in the way autoinducers, transcriptional factors and gene promoters interact with each other. A better understanding of these mechanisms is therefore crucial to evaluate the specificity of the system. Given the large variety of acyl-homoserine lactone derivatives which can serve as QS signals, it may also represent a privileged opportunity to get insight into possible crosstalk between different bacterial QS or to develop strategies of quorum-quenching. By combining biochemical and structural approaches with analysis of mutant strains and *in vivo* expression assays, the investigations on *A. tumefaciens* QS undoubtedly assemble one of the most elaborate sets of data in this domain.

### TraI AND OC8HSL SYNTHESIS

To identify the substrates of OC8HSL synthesis, the enzymatic activity of a purified *A. tumefaciens* TraI protein was tested in the presence of different molecules (More et al., 1996). It was thus determined that 3-oxo-octanoyl-acyl carrier protein (OC8-ACP) was the fatty acid donor and *S*-adenosylmethionine (SAM) the homoserine lactone precursor involved in OC8HSL synthesis. Mechanistically the synthesis reaction is proposed to occur in a “bi-ter” (two substrates, three products) way. The donation of the 3-oxo-octanoyl branch to the amine of SAM leads to the releases of first apo-ACP, then OC8HSL and finally methylthioadenosine (Parsek et al., 1999). All enzymes of the LuxI family are expected to share similar mechanisms of reaction, though variations in the acyl chain length and oxidation state at C3 of their acyl-ACP substrates exist. High-resolution crystal structures were obtained for two TraI orthologs: EsaI of *Pantoea stewartii* that synthesizes 3OC6HSLs and LasI of *Pseudomonas aeruginosa* that synthesizes 3-oxo-dodecanoyl-homoserine lactones (Watson et al., 2001; Gould et al., 2004). Analyses of these structures revealed that conserved residues in the N-terminal part of the protein were essential for SAM-binding and that selectivity of the acyl-ACP substrate was dependent on a V-shaped cleft passing through the enzyme. Other results also suggested that selectivity of LuxI-like proteins could be affected by availability of different acyl-ACP substrates. Noticeably, besides OC8HSL, *A. tumefaciens* produces traces of OC6HSL and octanoyl-homoserine lactone (C8HSL; Zhu et al., 1998).

### OC8HSL SPECIFICALLY INTERACTS WITH TraR

**FIGURE 3:**
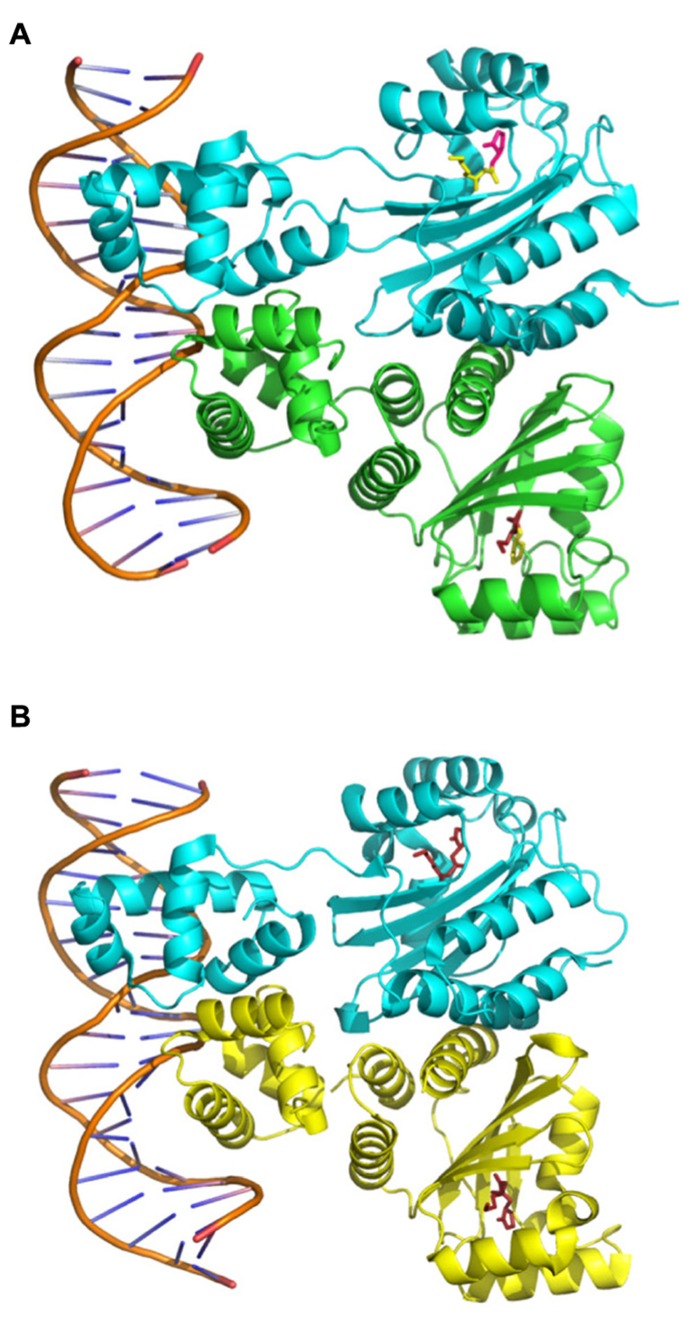
**Structures of the TraR–OC8HSL dimers in complex with DNA.** The images were created using data from The Protein Data Bank (PDB; www.rcsb.org) (Berman et al., 2000) and the PyMOL Molecular Graphics System software. **(A)** PDB ID: 1H0M from Vannini et al. (2002). **(B)** PDB ID: 1L3L from Zhang et al. (2002b).

The first evidence of the interaction between TraR and OC8HSL was obtained through purified active TraR complexes which co-eluted with OC8HSLs in a ratio 1:1 (Zhu and Winans, 1999). Analysis of the protein turnover also indicated that binding of OC8HSL occurs rapidly in cells, surely during the own synthesis of TraR on polysomes (Zhu and Winans, 2001). Further crystal structures provided a mechanistic explanation for the specific interaction between TraR and OC8HSL as they revealed that the N-terminal part of TraR formed an enclosed cavity into which OC8HSL molecule could be engulfed and tightly maintained through numerous hydrophobic interactions as well as four hydrogen bounds (Vannini et al., 2002; Zhang et al., 2002b; **Figure 3**). To analyze the specificity of the interaction between OC8HSL and TraR, 31 analogs of OC8HSLs were tested for their abilities to activate TraR. Most of these compounds turned out to be potent antagonists of TraR under wild-type conditions of TraR expression and significant stimulators under conditions of TraR overexpression. These two features demonstrate that the specificity of the interaction between TraR and its ligand could be dependent on TraR concentration (Zhu et al., 1998). Moreover the 3-oxo function of the OC8HSL molecule seems to play important role in the interaction process as 3-oxo-C6-, 3-oxo-C7-, 3-oxo-C11-, and 3-oxo-C12-homoserine lactones (see structures in **Figure 1**) can also activate TraR, though with a much lower intensity than OC8HSL (Zhu et al., 1998; Luo et al., 2003b). Consistently non-conservative mutations of the threonine 129 of TraR, that was predicted to stabilize the 3-oxo group in the binding pocket, led to a strong impairment of TraR activity (Chai and Winans, 2004). In addition, alanine 49 and glutamine 58 in the N-terminal part of TraR were found to be important for the binding of the C8 acyl chain of OC8HSL since their conversion to bulkier amino acids resulted in higher affinity toward homoserine lactone derivatives with shorter acyl chain (Chai and Winans, 2004).

### INTERACTION BETWEEN OC8HSL AND TraR FACILITATES FORMATION OF ACTIVE HOMODIMERS

The observation that C-terminal deletion mutants of TraR exerted strong dominant negativity over their wild-type counterparts led to the hypothesis that TraR–OC8HSL complexes had to multimerize to be active (Luo and Farrand, 1999). Thereafter, size exclusion chromatography techniques revealed that purified active OC8HSL–TraR complexes formed homodimers, and hybrid expression reporter systems demonstrated that OC8HSL was required for this dimerization to take place (Qin et al., 2000). The existence of active OC8HSL–TraR homodimers was further supported by analysis of crystal structures which also suggested that these dimers were significantly asymmetric (Vannini et al., 2002; Zhang et al., 2002b). Two dimerization domains were identified in TraR sequence, one in the N-terminal part of the protein, partially overlapping with the OC8HSL-binding domain and another, less extensive, in the C-terminal part (Luo et al., 2003a). Several findings illustrated the role of OC8HSL binding in the maturation and dimerization process of TraR. In absence of OC8HSL, TraR proteins were intrinsically unstructured, insoluble in cells and rapidly degraded by proteases. On the opposite, presence of OC8HSL directed the release of active TraR into cytosol and enhanced the resistance of the protein against proteolysis (Qin et al., 2000; Zhu and Winans, 2001; Pinto and Winans, 2009). Additionally the proper folding of TraR and acquisition of mature ternary structure following the interaction with OC8HSL was shown to be mediated by the chaperone GroESL (Chai and Winans, 2009).

### TraR–OC8HSL HOMODIMERS SPECIFICALLY RECOGNIZES tra BOXES

As mentioned above, tra boxes are 18 bp-inverted repeat operator sequences with a pronounced dyad symmetry, found in the two classes of TraR-regulated promoters (Fuqua and Winans, 1996a). The crystallization of TraR–OC8HSL complexes in presence of the tra box I sequence strongly suggested that each subunit of TraR–OC8HSL dimer binds to half of the tra box via C-terminal helix-turn-helix DNA binding motifs, thereby leading to an extensive DNA–protein interaction (Vannini et al., 2002; Zhang et al., 2002b; **Figure 3**). However, it was later demonstrated that six nucleotides at the center of the tra boxes did not interact with TraR and that yet these nucleotides contributed to proper activation of transcription, presumably by creating a flexible DNA bend (White and Winans, 2007). In parallel different screenings of TraR mutants resulted in the identification of three regions located in the N- and C-terminal part of the protein, which are critical for transactivation function but not for accumulation or DNA binding ability (Qin et al., 2004a, 2009; White and Winans, 2005). This finding suggested that these regions could cooperatively modulate the recruitment of the RNA polymerase and thereby differently control the expressions of TraR-regulated genes. Consistently some TraR mutants defective in transactivation of the *traI* promoter could still activate the *traM* promoter (Costa et al., 2009).

### TraM-MEDIATED INACTIVATION OF TraR IS DUE TO OLIGOMERIC ASSOCIATION

In an effort to better understand how TraM could deactivate TraR, two crystal structures of TraM were obtained. They showed that the TraM protein can form homodimers with one unit linked to the other by an extensive hydrophobic interface (Chen et al., 2004; Vannini et al., 2004). The importance of this interface and the dimerization properties of TraM were also assessed using deletion mutants (Qin et al., 2004b). In addition, purifications of inactive TraR/TraM complexes carried out by different groups and with different biochemical techniques led to the conclusion that the inactive complexes were composed of two TraR–OC8HSL dimers and two TraM dimers both *in vitro* and *in* *vivo* (Chen et al., 2004; Vannini et al., 2004; Qin et al., 2007). Several domains important for this oligomerization and the resulting inhibitory effect were identified both in TraR and TraM sequences (Luo et al., 2000; Swiderska et al., 2001; Qin et al., 2007). Moreover, to explain the way TraM could inactivate DNA-bound TraR–OC8HSL dimers, a study convincingly proposed a stepwise mechanism according to which the apparition of inactive TraR–OC8HSL/TraM complexes was preceded by a nucleoprotein intermediate comprising one dimer of each protein in association with DNA (Qin et al., 2007). Interestingly the biochemical and structural properties of the TraR/TraM complexes were also investigated in the *Rhizobium* sp. strain NGR234 and led to similar conclusions regarding the mechanisms by which TraM can negatively impact TraR functions (Chen et al., 2007).

## PLANT FACTORS ASSOCIATED TO *A. tumefaciens* QS

### ROLE OF THE OPINES: MASTER CONTROL AND FINE-TUNING OF QS REGULATION

Opines are the small organic compounds which are produced during development of crown gall disease in transformed plant cells through the action of synthesis genes present on the T-DNA. All *A. tumefaciens* Ti plasmids harbor operons specialized in the uptake and assimilation of the opines they contribute to produce (Dessaux et al., 1992, 1998; Platt et al., 2012b). The two types most investigated in laboratories are the octopine- and the nopaline-type. Moreover, specific opines, called conjugal opines, are strictly required to enable conjugation of the *A. tumefaciens* Ti plasmid (Kerr et al., 1977; Petit et al., 1978). Therefore the finding, at the beginning of the 1990s, that this phenomenon was also dependent on the TraR/TraI QS system (Zhang and Kerr, 1991), sparked off significant interest and a number of studies aimed at understanding how these regulatory steps could be related. Successive genetic analysis, sequence determination and promoter dissections ultimately allowed the complete elucidation of the signaling pathway, clearly establishing the prominent role played by the conjugal opines for *traR* expression and QS initiation.

In the case of nopaline-type Ti plasmids, agrocinopines A and B which are a mixture of two non-nitrogenous phosphodiesters of sugars serve as conjugal opines (Ellis et al., 1982). These molecules can provoke, presumably by direct inhibitory interaction, the release of the transcriptional repression exerted by AccR, a member of the FucR family of transcriptional regulator (Beck von Bodman et al., 1992). In turn this derepression causes the expression of two divergently oriented operons: the *acc* (agrocinopines catabolism) and *arc* (agrocinopine regulation of the conjugation) operons of the Ti plasmid. The *acc* operon encodes seven proteins involved in internalization and degradation of agrocinopines plus the repressor AccR (Kim and Farrand, 1997) while the *arc* operon encodes five proteins, the fourth being TraR (Piper et al., 1999). In contrast, in octopine-type Ti plasmids, *traR* is the last of 14 genes of the *occ* operon which codes for functions associated with octopine assimilation (Fuqua and Winans, 1996b). Octopine molecules are formed in transformed plant cells from arginine and pyruvate. Octopine is a conjugal opine as it binds to OccR, a transcriptional activator of the LysR family, thereby eliciting transcription of the *occ* operon including *traR* (Habeeb et al., 1991; Cho and Winans, 1993). Remarkably, the absence of the conjugal opines totally prevents QS-mediated conjugation of both nopaline- and octopine-type Ti plasmids. Moreover, despite the differences in *traR* location, the structures of the TraR-regulated operons are well conserved between the nopaline- and octopine-type Ti plasmids (Cho and Winans, 2007). This feature actually supports the view that *traR* and TraR-regulated genes constitute a functional unit, subjected to multiple and fortuitous recombination events in the course of *A. tumefaciens* evolution, and whose integration under the strict control of an opine regulon may have resulted in an important selective advantage for the bacteria (Piper et al., 1999; Oger and Farrand, 2001). In this sense the fact that such different molecules as agrocinopines and octopine can regulate *traR* expression in different Ti plasmids is remarkable.

Apart from the master control depicted above, opines are also involved in at least two other fine-tuning QS regulatory mechanisms. The first one was described in the *A. tumefaciens* strain R10 that harbors an octopine-type Ti plasmid. In this strain, the existence of a TraR antiactivator encoded by the Ti plasmid and different from TraM, named TrlR, was evidenced. Interestingly, TrlR expression was inducible by the opine mannopine (Oger et al., 1998). TrlR strongly resembles TraR but lacks its DNA-binding domain (Zhu and Winans, 1998). Experimental data provided evidence that TrlR could block TraR activity by forming inactive TrlR:TraR dimers (Chai et al., 2001). However, the impact of TrlR on QS implementation, especially *in vivo*, remains poorly understood. A second example of QS fine-tuning by opines is documented. In the nopaline-type *A. tumefaciens* C58 strain, expression of the Ti plasmid gene *aiiB* was shown to be induced by the agrocinopines, the same opines which are required for QS initiation (Haudecoeur et al., 2009b). Curiously *aiiB* codes for the AiiB lactonase that is highly similar to the AiiA lactonase from *Bacillus* sp. These proteins belong to a large family of Zn-hydrolases that encompasses lactonases of *Arthrobacter*, *Bacillus*, *Klebsiella*, *Mesorhizobium*, *Photorhabdus*, and *Rhizobium*. Biochemical and structural properties of AiiB were investigated. The AiiB protein is able to cleave the lactone rings of a large range of homoserine lactone derivatives, with a general preference for non-3-oxo-substituted molecules and substrates with an acyl chain longer than four carbons (Liu et al., 2007). Further conjugation experiments demonstrated the capacity of this lactonase to modulate *A. tumefaciens* QS responses both *in vitro* and *in planta* (Haudecoeur et al., 2009b). Globally the characteristics of *trlR* and *aiiB* (specific to octopine- and nopaline-type, respectively, and close homologs to *traR* and *aiiA*, respectively) suggest that these two genes could have arisen from gene duplication (for *trlR*) and horizontal gene transfer (for *aiiB*). On the other hand the conservation of an opine dependent regulation of their expression implies that there would be – somehow paradoxically – an advantage for *A. tumefaciens* cells to dampen QS communication at moments when opines, including conjugal opines, accumulate in tumors.

### THE EXPRESSION OF THE OC8HSL-DEGRADING BlcC (FORMERLY AttM) LACTONASE IS INDUCED BY PLANT METABOLITES

As AiiB, the BlcC protein is a member of the AiiA lactonase family. Different studies have shown that BlcC degrades various homoserine lactone derivatives, including gamma-butyrolactone (GBL, see structure in **Figure 1**) and OC8HSLs. The *blcC* gene is part of the three-gene *blcABC* operon which codes for the catabolic pathway converting GBL to succinate, through gamma-hydroxybutyrate (GHB) and succinic semialdehyde (SSA) intermediates (Chai et al., 2007). Remarkably BlcC confers to *Agrobacterium* the ability to grow with GBL as sole source of carbon, but it does not with OC8HSLs (Carlier et al., 2004). The expression of the *blcABC* operon is tightly controlled by the transcriptional repressor BlcR. Carbon and nitrogen starvation, GBL, GHB, and SSA can all release the repression exerted by BlcR, hence allowing the expression of the *blcABC* genes (Zhang et al., 2002a; Carlier et al., 2004). The plant metabolite gamma-amino butyric acid (GABA), through conversion to SSA (Chevrot et al., 2006; Wang et al., 2006b), and the plant defense signaling hormone salicylic acid, through an unknown mechanism (Yuan et al., 2008), can also induce *blcC* expression. Based on the observations that GABA induces the expression of the *blcABC* operon and that GABA accumulates in tumors, it was proposed that the BlcC activity could coincide with QS communication during interactions between *A. tumefaciens* and plant hosts. However, in tomato tumors, the effect of BlcC on QS-dependent Ti plasmid conjugation was weak and transient (Khan and Farrand, 2009), suggesting that plant tumor tissues could exert a negative control on the expression of the BlcC expression.

The capacity of *A. tumefaciens* to take up GABA was extensively investigated in the last years. Studies revealed the involvement of two distinct transport systems. The gene *atu2422*, located on the circular chromosome is widely conserved within the *Agrobacterium* genus and codes for a periplasmic GABA-binding protein that controls GABA import through the *bra* ABC transporter (Planamente et al., 2010). Interestingly the GABA import by *atu2422* is strongly antagonized by proline, alanine, and valine, suggesting that these compounds which accumulate in tumors could also indirectly modulate the overall BlcC lactonase activity in the bacterial cells (Haudecoeur et al., 2009a). In comparison, the periplasmic binding protein encoded by the linear chromosome gene *atu4243* appears highly specific for GABA (Planamente et al., 2012). Strikingly, the expression of *atu4243* is totally repressed by *atu4232*-encoded protein and mechanisms of derepression are so far unknown (Planamente et al., 2012). Collectively these data illustrate the complexity of factors coming into play when searching to determine the impact of BlcC on *A. tumefaciens* QS. Of special interest would be the critical examination of plant metabolism to evaluate how the GABA, GBL, GHB, and SSA produced in the tumors may activate BlcC in colonizing *A. tumefaciens* cells. Such studies might reveal that the role of BlcC varies according to the metabolic status of the plant hosts.

### INTERACTIONS BETWEEN THE Ti AND AT PLASMIDS IN THE PLANT TUMOR

Another interesting feature of the *blcC* gene lies in its location on the companion At plasmid. This makes it the only component involved in *A. tumefaciens* QS that is not present on the Ti plasmid. Ecologically this characteristic raises interesting questions and notably that to know whether the dissociation of the At and Ti plasmids could result in a QS deregulation. To date very little is known about the maintenance of the At plasmid in *A. tumefaciens* populations. If no gene essential for the survival of *A. tumefaciens* C58 is present on the At plasmid (Goodner et al., 2001; Wood et al., 2001), the carriage of this At plasmid imposes *in vitro* high fitness costs to *A. tumefaciens* host cells (Morton et al., 2013). On the other hand, the At plasmid encodes several functions which confer or may confer a fitness advantage to agrobacteria in plant tumors (Haudecoeur et al., 2009b). Besides the degradation of butyrolactones and their derivatives mentioned above, the At plasmid is involved in the assimilation of some opines of Amadori compounds (Vaudequin-Dransart et al., 1998; Baek et al., 2005). The At plasmid also seems to have a positive impact on the virulence capacity of *A. tumefaciens* (Matthysse et al., 2008), although this point is debatable as it was recently shown that a large deletion in the At plasmid resulted in increase of the bacterial virulence (Morton et al., 2013). In conclusion, one can reasonably assume that, as for Ti plasmids, the tumor compartment is an appropriate environment for the dissemination of the At plasmid. Remarkably it was recently demonstrated that in *A. tumefaciens* C58, the conjugations of At and Ti plasmids are related events controlled by the agrocinopines-responsive regulator AccR and it was suggested that this mechanism of co-regulation could be instrumental in the conservation of the reciprocally beneficial functions carried by the two replicons (Lang et al., 2013).

### OC8HSL-ASSOCIATED PLANT RESPONSES

The interactions between *A. tumefaciens* and plant hosts are mediated by several factors, from the phenolic compounds accumulated at wound sites that induce the expression of the Ti plasmid *vir* genes, to the opines produced in the tumor niche that control horizontal transfer of bacterial plasmids. It is therefore tempting to speculate on a possible implication of QS signal molecules in this generic trans-kingdom association, especially as several lines of evidence showed that *N*-acyl-homoserine lactone molecules could induce specific responses in eukaryote cells (Williams, 2007). For instance, in axenic plant systems, exogenous supply of different homoserine lactone derivatives was found to modulate plant immunity and development although the outcomes drastically differed according to the nature of the tested QS molecules (Klein et al., 2009; Hartmann and Schikora, 2012).

To our knowledge only three studies investigated the impact of OC8HSL on plants. In the first one, authors devised an inducible gene expression system based on TraR-OC8HSL activity which they introduced in *Arabidopsis thaliana* plants (You et al., 2006). To verify that induction with OC8HSL of the transferred gene did not affect the transcriptome of the transformed plants, the authors extracted RNA from 12-day-old seedlings treated or not by foliar application with 1 mM of OC8HSL for 24 h and carried out microarray experiments using Agilent technology. Processing of the data prompted them to conclude that no gene was differentially expressed by presence of the QS signal. In a second paper, a proteome analysis of *Arabidopsis thaliana* roots grown for 24 h in a hydroponic system in the presence or not of 10 μM of OC8HSL revealed that the levels of 53 proteins involved in the metabolism of carbohydrate and energy, protein biosynthesis, defense responses, and cytoskeleton remodeling, were significantly affected by the QS signal (Miao et al., 2012). The modest number of proteins differentially affected in this study suggests that plants sense *A. tumefaciens* QS signals only in a very restricted way. Noteworthy, in the two above-mentioned experiments, the used concentrations of homoserine lactone derivatives were in the micromolar and millimolar range while the concentrations at which QS molecules are active in *A. tumefaciens* are usually rather in the nanomolar range. Finally *Arabidopsis thaliana* defense responses upon exposure to OC8HSL-producing *Rhizobium etli* were recently analyzed. The results established that this condition had no impact on the plant defense (Zarkani et al., 2013), thereby strengthening the notion that plants are immune to OC8HSLs.

## IMPLICATIONS AND SELECTIVE ADVANTAGES OF THE TIGHTLY REGULATED QS SYSTEM IN *A. tumefaciens*

**FIGURE 4:**
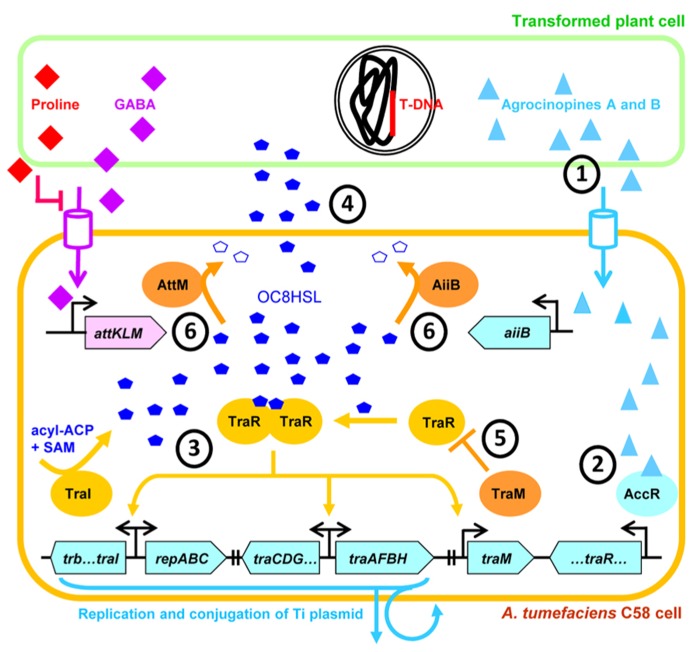
**Representation of the sophisticated hierarchical QS regulation in *A. tumefaciens* strain C58.** QS-dependent conjugation and copy-number amplification of the Ti plasmid is initiated when OC8HSL and TraR reach appropriate concentration and form transcriptional active dimers. QS-signaling is positively regulated by the conjugal opines agrocinopines which are produced by the transformed plant cells **(1)** and induce production of TraR by releasing AccR repressive action **(2)**. Then, active TraR-OC8HSL dimers activate the production of TraI, thereby triggering a positive feedback in the synthesis of OC8HSLs **(3)** which are diffusible molecules **(4)**. The QS activation is delayed by the TraR-antagonist TraM **(5)**, as well as OC8HSL-cleaving lactonases AiiB and AttM **(6)** whose expressions are controlled by agrocinopines and GABA, respectively. SAM, *S*-adenosylmethionine; ACP, acyl carrier protein. The Ti plasmid genes and the At plasmid genes are in blue and pink boxes, respectively.

Taken together the findings presented above described a very sophisticated system in which *A. tumefaciens* QS action is not only placed under the strict control of the conjugal opine regulon but is also modulated by various adjacent components like antiactivator or lactonases (**Figure 4**). Now we will discuss the implications of such hierarchical regulatory cascades and speculate about the selective advantages they may confer to *A. tumefaciens*.

### CONJUGATION OF Ti PLASMID IN OPINE-PRODUCING TUMORS

As mentioned previously, the expression of *traR* gene requires the presence of conjugal opines. Therefore the QS system of *A. tumefaciens* functions only in host plants and only after transformed tissues have accumulated sufficient amount of conjugal opines. This restriction suggests that mature tumors are the most conducive environments for Ti plasmid dissemination and that, in these plant tumors, the selective advantages conferred to *A. tumefaciens* by a functional Ti plasmid would overcome the associated costs of maintenance. Supporting these notions, it has been demonstrated that Ti plasmid imposed a high fitness cost under conditions reminiscent of tumorigenesis but not anymore when opines were fully supplied (Platt et al., 2012a). It has also been observed that large proportion of *A. tumefaciens* cells present in mature tumors were devoid of Ti plasmids or harbored a mutated Ti plasmid (Fortin et al., 1993; Belanger et al., 1995). Thus the master control by conjugal opines could allow a large dissemination of functional Ti plasmids in an *A. tumefaciens* population characterized by a high proportion of potential recipient cells. The resulting selective advantages would be manifold. By amplifying the number of genes involved in opine assimilation, this mechanism could increase the colonizing fitness of the *A. tumefaciens* population, especially in older tumors where nutritive resources are scarcer. Multiplication of *vir* genes may also enhance aggressiveness of the bacteria. In relation, several reports already correlated an impairment of *A. tumefaciens* QS communication with a diminution of the crown gall symptoms (Haudecoeur et al., 2009b; Planamente et al., 2010, 2012). At last dissemination of Ti plasmids would increase the potential of migratory agrobacterial cells to initiate new infections. Interestingly Ti plasmid transfers to other bacterial species present in plant tumors may also occur, a feature that would favor genetic biodiversity. In this regard it is unfortunate that, even if the plant tumors are generally considered as privileged entry points for other bacteria, no information on plant tumor microbiomes are available at the moment.

### DOES *A. tumefaciens* QS REALLY MEASURE A QUORUM OF DONOR CELLS?

Since the finding that *A. tumefaciens* QS controlled Ti plasmid conjugation, a “nagging” question remained to understand the relevance of a system in which donor cells could only monitor the density of other donors that already harbor a Ti-plasmid. Indeed as conjugation cannot happen in a cell already containing a resident Ti plasmid (Cho et al., 2009), the risk of uselessly activating, at the quorum concentration, the horizontal transfer machinery in the absence of sufficiently numerous recipient cells seems elevated. Nonetheless, as evoked previously, the master control of QS by conjugal opines might provide a way to circumvent this difficulty by allowing the conjugation of Ti plasmid only in mature tumors, i.e., in environments where the proportion of recipient cells would have extended. In such a context, the adjustment of the activation of the *tra* regulon according to a quorum of donor cells should maximize the efficiency of Ti plasmid dissemination and would be fully sensible. Under laboratory conditions, all the collected data firmly sustain the notion that *A. tumefaciens* QS functions as a cell density-dependent process. However, these conditions, using most of the time cell cultures and constant concentration of conjugal opines to initiate QS, may not reflect natural conditions. In *V. fischeri* the quorum nature of the system is defined by a production of LuxR at relatively high basal level and by a concentration of OC6HSL which increases as a function of cell density until reaching the threshold of LuxR activation (Miller and Bassler, 2001). In contrast, in *A. tumefaciens*, production of an active TraR regulator is subordinated to the presence of conjugal opines and to that of the antiactivator TraM. Taking full consideration of this characteristic implies that QS can be partly dissociated from solely functioning as a measure of population density. Another element of complexity may be brought by the non-linear accumulation of OC8HSL in tumors. Indeed plant tumors are not homogenous structures; they emerged from wound sites and underwent neoplastic expansion (Aloni et al., 1995; Veselov et al., 2003). In these complex environments colonizing *A. tumefaciens* shall form different clusters of cells more or less isolated one from the other and located in surface or intercellular spaces where diffusion rates are different as well as temporally changing. It therefore appears unlikely that the OC8HSL concentration which can be measured in a tumor or a part of the tumor does strictly mirror the cell density of the pathogen in this environment. Interestingly when they simulated the QS-induced transition in liquid cell cultures or biofilm, Goryachev et al. (2005) noticed that the first condition required a much higher threshold density than the second. They consequently came to the conclusion that *A. tumefaciens* QS served as a detector of biofilm formation rather than a sensor of cell concentration. If a growing attention has been given in the last years to mechanisms of biofilm formation in *A. tumefaciens* (Tomlinson et al., 2010; Hibbing and Fuqua, 2012), no data so far have related them to QS and very little is known about the formation of biofilms in the context of the agrobacterial interactions with plant host. However, it would definitely be relevant for the bacteria to place the coordination of Ti plasmid conjugation upon biofilm perception since the cell aggregates would constitute a very appropriate context for activation of the horizontal transfer machinery, either by minimizing the distances between donor and recipient cells or by acting as a shield against all kinds of physical or biological perturbations.

### RELATIONSHIP BETWEEN QS REGULATION, Ti PLASMID CONJUGATION, AND *A. tumefaciens* HOST CELL

In the above discussion, the question of the QS-dependent dissemination of Ti plasmids was addressed only according to the selective advantages this dissemination may confer to agrobacterial cells. However, another perspective would be to consider Ti plasmids as selfish elements which somehow hijack *A. tumefaciens* cells in order to disseminate their genetic backgrounds. In this framework Ti plasmids would take advantage of the opine and QS regulations to optimize the efficiency of their conjugations. It is furthermore important to note that the tumor conditions where the selective advantage conferred to *A. tumefaciens* cells by the Ti plasmids is the strongest coincide with the conditions where the dissemination of these Ti plasmids is the most important. The recent discovery in *A. tumefaciens* C58 that the conjugations of both Ti and At plasmids are exacerbated by conjugal opines (Lang et al., 2013) further supports the notion that Ti and At plasmids may collaborate to transform avirulent *A. tumefaciens* cells into virulent in order to perpetuate and disseminate their genetic traits.

## CONCLUSION

In this review, we described the *A. tumefaciens* TraI/TraR QS system and showed how it exquisitely regulated the dissemination of Ti plasmids.

The QS systems of LuxI/LuxR type are generally thought to have originated early in evolution of Gram-negative Proteobacteria, with functional pairs of autoinducer synthases and receptors coevolving as regulatory cassettes, although in many cases these cassettes could also be inherited horizontally (Gray and Garey, 2001). In *A. tumefaciens*, the TraI/TraR system and the related QS-regulated genes are well conserved in all nopaline- and octopine-type strains studied to date, suggesting that this regulatory mechanism has been anciently selected. The target genes of *A. tumefaciens* QS are involved in the dissemination of Ti plasmids, both by replication and conjugation, but also in positive and negative feedback controls with the OC8HSL-synthesis TraI enzyme and the TraM antiactivator. Different studies demonstrated that this last protein plays a critical role in the implementation of the QS, even if it is not clear yet whether TraM is more relevant in delaying QS activation or in stabilizing and limiting QS activity.

At the molecular level, the *A. tumefaciens* QS communication has been largely deciphered. Two crystal structures have notably been obtained for TraR, in association with OC8HSL and DNA, providing a first class access to the interaction specificities of the system. Thorough biochemical investigations of active and inactive complexes also allowed to better understand multimerization processes of the QS components.

Consistent with the particular phytopathogenic lifestyle of the bacteria, *A. tumefaciens* QS system displays an original scheme including several differently acquired regulatory elements. The most important of these elements, common to all *A. tumefaciens* strains, are the conjugal opines which accumulate in tumors as a consequence of plant transformation and are strictly required for *traR* expression and hence for QS initiation. In parallel, only specific to some *A. tumefaciens* strains, lactonases such as AiiB and BlcC or supplementary anti-activator like TrlR can also modulate QS responses. This complex network of horizontal and lateral regulation suggests that there would be an advantage for *A. tumefaciens* to restrain as much as possible the window of QS activation.

Assessing reasons why a biological system has been selected is always challenging because this selection hinges on a trade-off between advantages and drawbacks which cannot be fully appreciated under laboratory conditions. By perusing different possibilities, we nonetheless hypothesized that the tight regulation of *A. tumefaciens* QS surely allowed the bacteria to disseminate the Ti plasmid in an environment where carrying the replicon would be clearly advantageous and at a moment when the energetic and physical factors would be ideal.

For the future, some important questions still remain to be answered to complete our understanding of *A. tumefaciens* QS functioning during the interactions with the host plant. For instance how do conjugal opines and TraM cooperate to produce active TraR-OC8HSL dimers? Precise dosage of conjugal opines in the course of tumor development as well as advances in knowledge of *traM* regulation might help solve this question. It would also be very interesting to better determine how the BlcC lactonase interferes with OC8HSL levels in tumors induced on different plants hosts and what are the ecological implications regarding horizontal transfers of both At and Ti plasmids. At last, analysis of bacterial populations found in natural tumors could deliver exciting results regarding abundance of potential Ti plasmid recipient cells. This kind of data might also unveil the extent of competition between the phytopathogen and other bacterial species present in plant tumors, hence leading to a novel appreciation of *A. tumefaciens* QS activity.

## Conflict of Interest Statement

The authors declare that the research was conducted in the absence of any commercial or financial relationships that could be construed as a potential conflict of interest.
